# Breast cancer: emerging principles of metastasis, adjuvant and neoadjuvant treatment from cancer registry data

**DOI:** 10.1007/s00432-022-04369-4

**Published:** 2022-12-20

**Authors:** Jutta Engel, Renate Eckel, Kathrin Halfter, Gabriele Schubert-Fritschle, Dieter Hölzel

**Affiliations:** Munich Cancer Registry (MCR), Ludwig-Maximilians-University (LMU), 81377 Munich, Germany

**Keywords:** Breast cancer, Tumor growth, Local recurrence, Positive lymph node, Metastasis, Adjuvant treatment, Survival

## Abstract

**Purpose:**

Growing primary breast cancers (PT) can initiate local recurrences (LR), regional lymph nodes (pLN) and distant metastases (MET). Components of these progressions are initiation, frequency, growth duration, and survival. These characteristics describe principles which proposed molecular concepts and hypotheses must align with.

**Methods:**

In a population-based retrospective modeling approach using data from the Munich Cancer Registry key steps and factors associated with metastasis were identified and quantified. Analysis of 66.800 patient datasets over four time periods since 1978, reliable evidence is obtained even in small subgroups. Together with results of clinical trials on prevention and adjuvant treatment (AT) principles for the MET process and AT are derived.

**Results:**

The median growth periods for PT/MET/LR/pLN comes to 12.5/8.8/5/3.5 years, respectively. Even if 30% of METs only appear after 10 years, a pre-diagnosis MET initiation principle not a delayed one should be true. The growth times of PTs and METs vary by a factor of 10 or more but their ratio is robust at about 1.4. Principles of AT are 50% PT eradication, the selective and partial eradication of bone and lung METs. This cannot be improved by extending the duration of the previously known ATs.

**Conclusion:**

A paradigm of ten principles for the MET process and ATs is derived from real world data and clinical trials indicates that there is no rationale for the long-term application of endocrine ATs, risk of PTs by hormone replacement therapies, or cascading initiation of METs. The principles show limits and opportunities for innovation also through alternative interpretations of well-known studies. The outlined MET process should be generalizable to all solid tumors.

**Supplementary Information:**

The online version contains supplementary material available at 10.1007/s00432-022-04369-4.

## Introduction

Biomedical research regularly increases the complexity of cancer. More and more details are emerging on the association with risk factors, the steps involved in carcinogenesis, and processes such as clonal evolution that ultimately result in genetically heterogeneous primary tumors (PTs) (Valastyan and Weinberg 2011; Hanahan and Weinberg 2011; Yates et al. 2017). Steps to MET or the MET-process (MET-P), are also becoming increasingly differentiated (Lambert et al. 2017; Peinado et al. 2017). By definition, secondary MET (sMET) are referred to as local recurrences (LRs), positive lymph nodes (pLNs), and distant METs. Primary and secondary BCs (1stPT/2ndPT) are included because these may also be prevented and have the same risk of initiating sMETs.

Even though the molecular processes involved in the disease course of PTs and sMETs are becoming increasingly complex, they can still be described with well-known parameters and a few principles of METs and their treatment. The aim of this article is to elucidate initiation, growth, survival, and treatment effects of BC and sMET with real world data from the Munich Cancer Registry (MCR) which are basic conditions, bottlenecks through which molecular hypotheses and scientific terms for prognosis and prediction have to go.

## Methods

MCR has data starting from 1978. It has been population-based for the currently underlying 4.9 million population since 1998, and is included in Cancer Incidence of Five Continents (Bray et al. 2017). Reliable retrospective data on changing adjuvant treatments (AT) and on locoregional disease manifestations from pathology reports such as hormone receptor (HR) status, tumor diameter (TD), number of pLN, Ki67 and contralateral PT are available. All death certificates from the region are included and provide an up-to-date follow-up.

In the case of cancer-related death, approximately 70% of cases had a documented MET. Four time intervals have been distinguished since 1978 and the time trends of successful ATs and changing progressions are analyzed. Despite missing values, it is robust data with 66.818 patients for the analysis of even small subgroups. Kaplan–Meier curves for the relative survival from diagnosis, for survival up to and after MET and with distributions of the MET-free survival time describe the relationships with prognostic and predictive factors. The relative survival is an estimate for tumor-specific survival and is calculated by dividing the overall survival after diagnosis by the survival observed in the general population with comparable age distribution.

METs account for the great majority of cancer-associated deaths, this is why this complex process needs to be better understood. With every millimeter of PT growth, further METs are initiated. The time of occult MET growth to PT diagnosis, the MET-free time up to MET detection and post-MET survival afterwards are estimated. So far, modeling has seldom been used in medicine to elucidate relationships. Processes are modeled with the distribution functions for the incidence of PTs and METs, the growth times and eradication rates. The effect of hormone therapy (HRT) with faster growth of prevalent PTs and a risk of new PTs or the long-term endocrine AT with a preventive and adjuvant effect are examples. Statistical analyses were performed by using SAS V 9.4 and R V 3.1.3.

## Results on initiation and growth of tumor foci

### Initiation of primary tumors and secondary foci

**Fig. 1:**
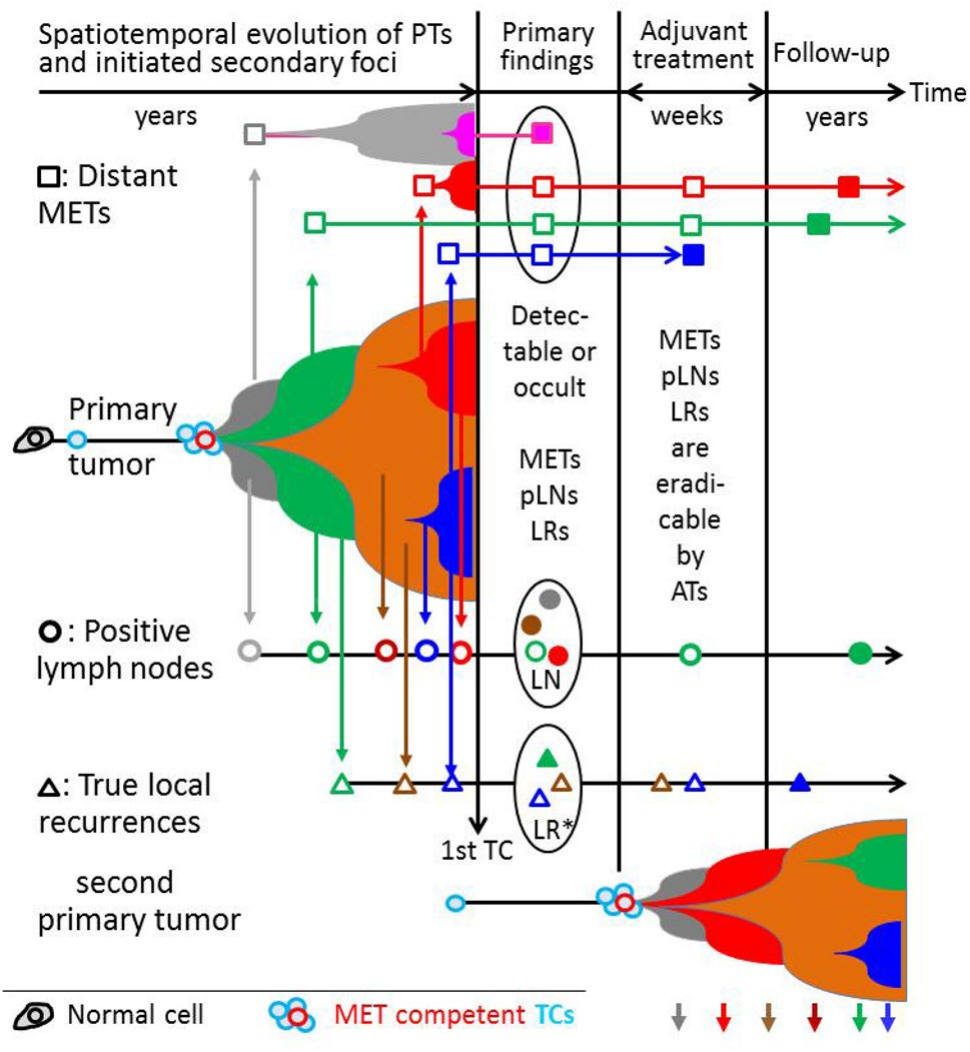
Initiation and growth of a PT and secondary foci. Growing PTs can initiate LRs, pLNs and METs with different gene signature. They can be diagnosed synchronously with PTs (filled symbols), remain occult, will be eradicated by ATs or occur in the course of disease. The article and this figure were inspired by LR Yates et al. (2017)

**Fig. 2:**
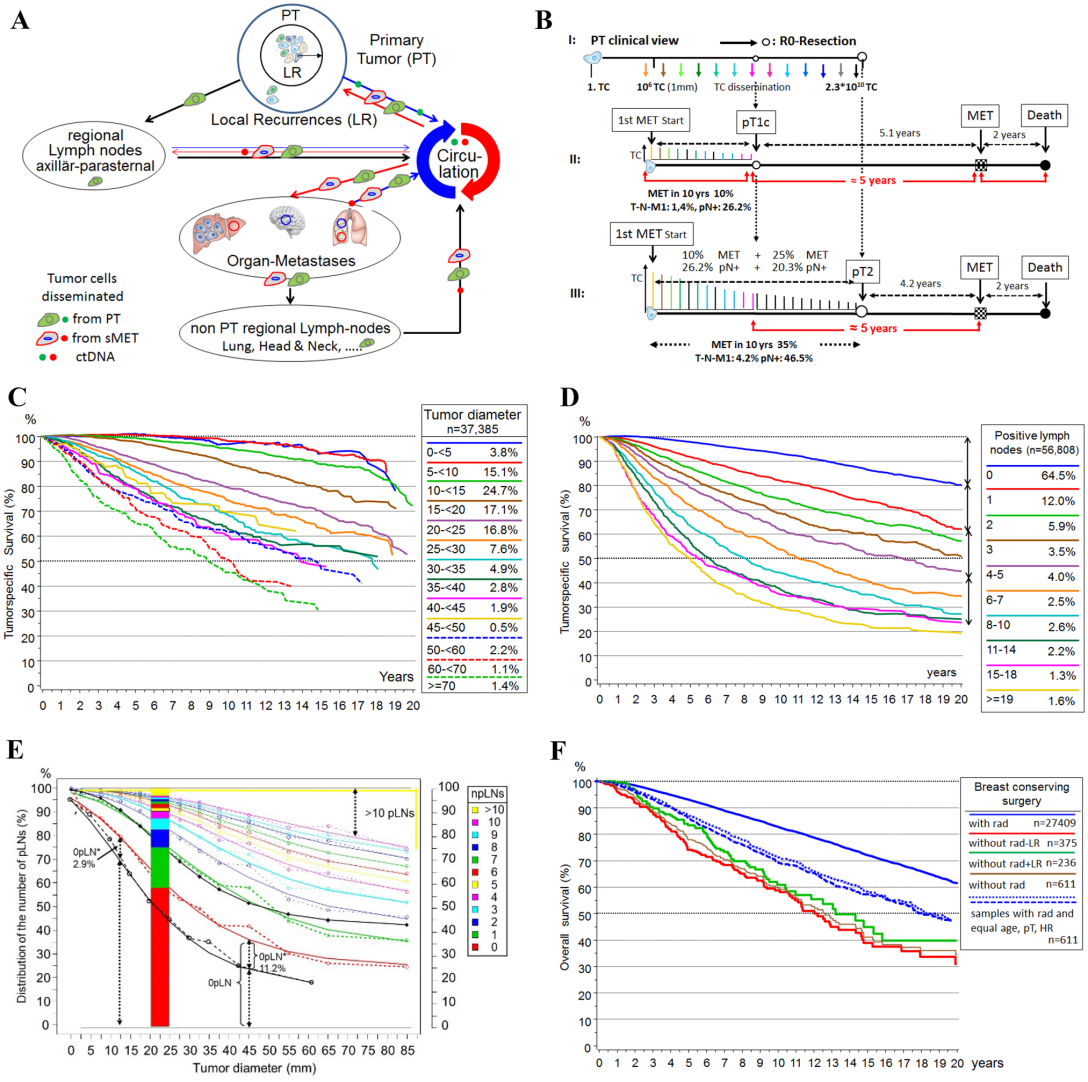

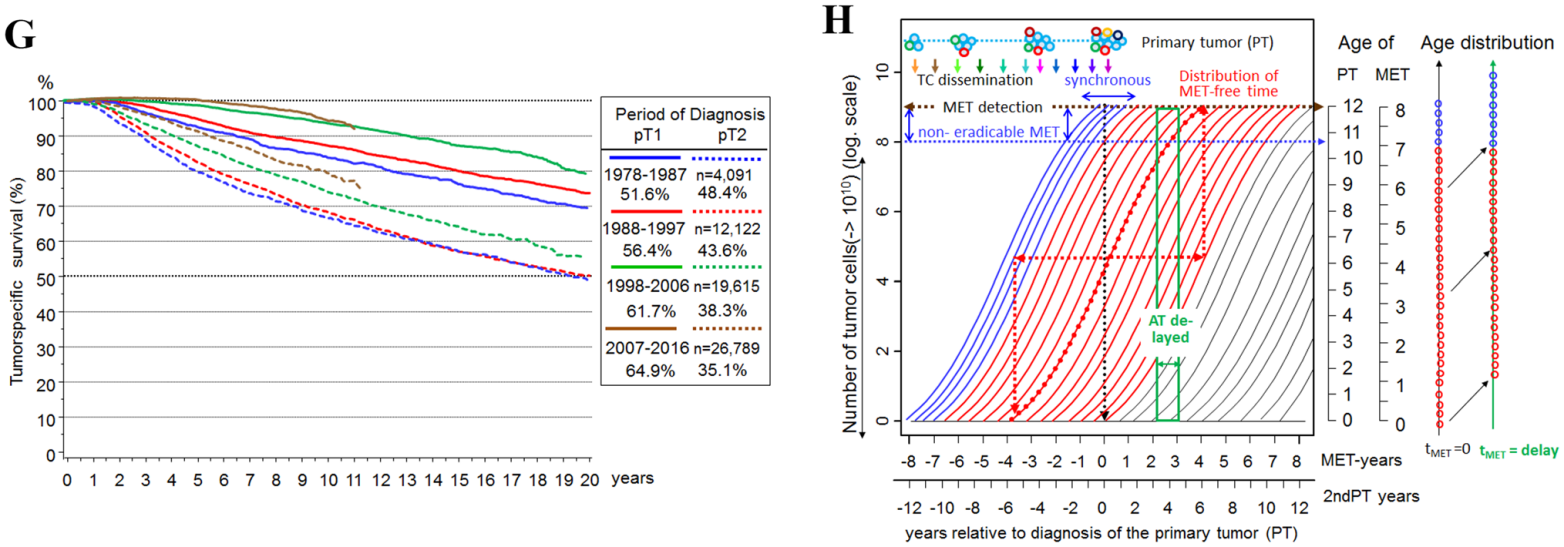
2A–2H combined. **A** Possible sources and pathways of tumor cell dissemination and initiation of LRs, pLNs, METs. **B** Tumor size, dependent MET and pLN initiation and survival. I: METs initiation until the removal of the PT. **C** Relative* survival depending on tumor diameter for T-N-M0 PTs. **D** Relative* survival depending on the number of pLNs (Engel et al. 2019). **E** Lymph node infiltration. Distribution of the number of pLNs in dependence on tumor diameter. **F** Overall survival for patients with pT1-2 PTs, breast conserving surgery and irradiation. **G** Relative* survival for pT1c and pT2 PTs and 4 time periods from 1978. **H** Growth trajectories for MET and 2ndPTs. *The relative survival is an estimate for tumor-specific survival and is calculated by dividing the overall survival after diagnosis by the survival observed in the general population with comparable age distribution

Growing and evolutionarily developing PTs may have already disseminated DNA (ctDNA) and circulating tumor cells (TC) to form local or distant small foci through alterations in the tumor microenvironment (Husemann et al. 2008; Narod and Sopik 2018). Several cell types and signaling molecules are involved in promoting the epithelial–mesenchymal transition that allow TCs to disseminate. PTs can achieve MET-competence starting from about 1 mm TD (Lambert et al. 2017) and initially a first MET-competent TC appears among many disseminated TCs (Butler and Gullino 1975). They may remain local or may spread through lymphatic or hematogenous dissemination and initiate LRs, pLNs, and distant METs. These sMETs grow in parallel and are usually discovered at the earliest with the PT diagnosis (Fig. 1). In Fig. 2A, possible sources and pathways of the initiating TCs are outlined and some results for pT1c- and pT2-PTs are arranged in Fig. 2B. Growing PTs are associated with worsening prognosis (Fig. 2C). Until R0-resection, all TCs that can initiate sMETs are disseminated. Only dormancy could delay the onset. The liquid biopsy concept with cTCs and ctDNA can be used to detect small foci early clinically and radiologically (Pantel and Alix-Panabières 2019; Menyailo et al. 2020; Cresswell et al. 2020; Bidard et al. 2014).

### Initiation of local recurrences

When TCs migrate but remain close to the PT, they can initiate true LRs. These usually occur within 3 cm (Fig. 2A), the target area of the boost irradiation. The shared microenvironment of the PT forms a supporting niche providing particularly favorable growth conditions. This was shown in historical data from the NSABP-B-06 study with 39.2% LRs after breast conserving surgery without irradiation (Fisher et al. 2002). However, the term LRs is inaccurate because LR such as METs or pLNs can also occur synchronously with PTs in about 18% and are then referred to as multifocal PTs.

In total, four types of LR can be distinguished: growing residual tumors from positive margins, true PT-near LRs, and independently initiated ipsilateral 2ndPT which, if synchronous, are called multicentric PTs (Fig. 1) (Panet-Raymond et al. 2011; Whipp et al. 2010). A fourth type may be ipsi- or contralateral foci that emerge through self-seeding (Comen and Norton 2012; Kim et al. 2009). Since these are initiated by TCs, which find their way back through circulation, they likely exhibit similar characteristics as true LRs but without the proximity to the PT (Fig. 2A). As data from the MCR shows, initiation of pLNs becomes more likely with about 15% more pLN findings for pT1-2 m (multiple) PTs (Brierley et al. 2016).

### Initiation of positive lymph nodes

TCs can infiltrate LNs through lymphatic dissemination. This LN infiltration is also a stochastic process over time, with the number of pLNs representing discrete successive steps (Engel et al. 2019). Figure 2B shows LN infiltration depending on pT-categories. In pT2-PTs more than half (26.2%) of the 46.5% pLNs have been infiltrated by TCs during growth up to pT1c. In the subgroup of HR + non-advanced PTs, the percentage of patients with more than npLNs can be described by Gompertz functions with 3 parameters (Fig. 2E). In addition, large PTs can develop the required driver mutations at a later point in time but the infiltration process is not significantly affected. As the TD increases, the fraction with > 10 pLNs increases at the expense of 0pLNs, the proportion of which varies between 0.05% and 1.75% per millimeter. However, the proportion with 1-2pLN is largely independent of the TD. In the 0pLN status, some of the PTs have begun to disseminate MET-competent TCs (Fig. 2D) and approximately 15.9% of isolated TC or micro-MET are detected, the proportion of which is shown as a function of diameter in Fig. 2E as the 0pN* line and corresponds to the proportion of 0pLN to 1pLN (Engel et al. 2019; Mamounas et al. 2017).

### Initiation of metastases

When are METs initiated? As described above, pT1-PTs show about 13% tumor-related deaths in the first 15 years (Fig. 2G). When comparing such results, the time reference of the cohort and therefore the ATs of the 2000s should be taken into account because progress has been made with successful innovations. If PTs are diagnosed later as pT2-PTs with a mean TD of 28 mm, the METs already initiated up to a tumor size of pT1c continued to grow and 25% new METs not eradicable by ATs are initiated after pT1c size (Fig. 2B) (Munich Cancer Registry 2021). In the pT2 category, the proportion of advanced PTs has already reached 4.2% and the MET-free interval shortens to 4.2 years. The time scale of the MET-P in pT2-PTs illustrates that advancing the PT diagnosis avoids METs and results in a longer MET-free interval of 5.1 years. This constitutes a lead time effect for the subgroup with unavoidable METs, which are initiated up to a tumor size of pT1.

The initiation of MET is similar to that of LNs. At some point, a TC succeeds in infiltrating a distant organ site, perhaps cooperatively as a homogeneous cluster or with other heterogeneous circulating TCs (polyclonal origin) (Keller and Pantel 2019). Therefore, METs are already initiated in the pN0 phase as shown by the doubling of mortality from 0 to 1pLN (Fig. 2D). Thereafter, additional METs can be initiated sequentially by a PT, which can acquire further mutations in the meantime (Fig. 1). Therefore, multiple METs in one or more organs and also in different pLNs can be genetically different (Yates et al. 2017; Turajlic and Swanton 2016; Desmedt et al. 2016). In addition, different areas of a PT can already be assigned as source of TCs (Yachida et al. 2010).

### Combinations of initiated sMETs

If a PT disseminates MET-competent TCs in principle all three sMETs can be initiated. LRs are more common than pLNs, which in turn are more common than METs because TCs have to overcome additional lymphatic and hematogenous barriers, extravasation and colonization into organs (Valastyan and Weinberg 2011). All combinations of sMETs in any order are possible. The competing initiation is illustrated in the 15-year relative survival which is 88.1/93.8/87.9/84.7% for pT1c and all/pN0/1pLN/2pLNs, respectively. The corresponding values for pT2 are 67.4/80.7/72.9 /64.6% and show the high proportion of pLNs and METs initiated early and growing in parallel with the PT. MET organ-specific properties of TCs may play a role in this process (Zhang et al. 2013; Bos et al. 2009; Weiss 1992).

### Initiation of primary tumors

The age-dependent incidence reveals the initiation of 1stPTs (Munich Cancer Registry 2021; Noone et al. 2021), that occurs years before according to the growth duration of the PTs. About 2% of patients have bilateral PTs which must be initiated nearly at the same time and then grew in parallel. The incidence of contralateral 2ndPT and PTs in prevention studies (Cuzick et al. 2015) show that year after year these PTs are diagnosed and must have been prevalent in the patient cohort according to the duration of the growth of PTs (Fig. 2H). In high-risk groups (Gail et al. 1989) for example with BRCA mutations the incidence increases to 60–80% compared to today’s lifetime incidence of approximately 12.4% in the normal population (Noone et al. 2021). In particular, BC patients have a three- to fivefold risk of developing 2ndPTs compared to the incidence of 1stPT in the normal population (Chaudary et al. 1984).

## Source and time of late MET initiations

### Dormant TCs

**Fig. 3:**
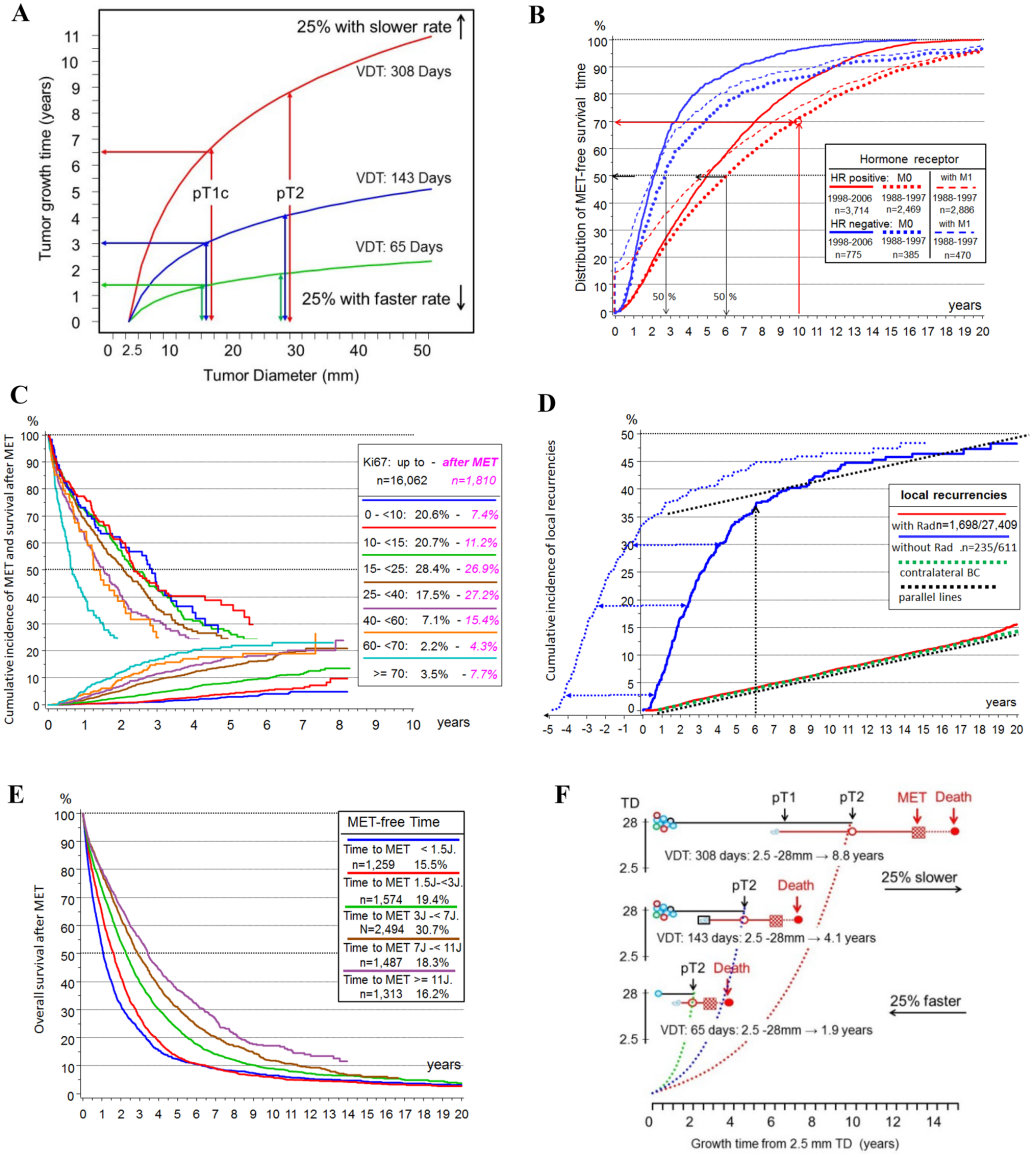

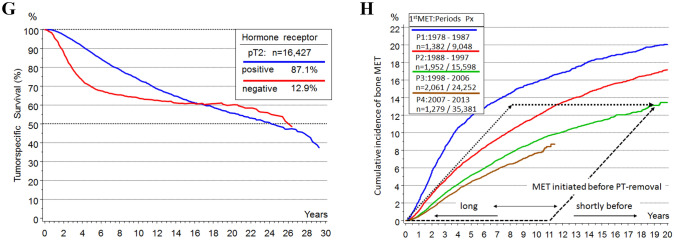
3A–3H combined. **A** Volume doubling and tumor growth. **B** Hormone receptor dependent MET-free survival. **C** Cumulative incidence of METs and survival after MET depending on KI67 **D** Cumulative incidence of LRs after breast conserving surgery with and without irradiation. **E** Overall survival from MET of HR + PTs as a function of MET-free time. **F** PT and MET growth relation principle. **G** Relative survival of HR + and HR- PTs. **H** Time trend of bone MET-free survival

DTCs could be an inexhaustible source to initiate MET after PT removal (Aguirre-Ghiso et al. 2013; Oskarsson et al. 2014; Meng et al. 2004). But the biology of these DTC and their natural history over a patient’s lifetime is largely unclear (Bushnell et al. 2021; Werner et al. 2021). The only argument for their existence is the detection of vital TCs in organs (Janni et al. 2011). Such TCs exist and are a prognostic factor but probably not a relevant cause of METs. Improved diagnostics reveal more synchronous LR and advanced PTs. More elaborate preparation of sentinel LNs detects isolated tumor cells. Occult METs of all sizes are also present in organs as the distributions of MET-free survival show (Fig. 3B). Late METs are initiated shortly before PT diagnosis and their MET-free periods of 10 and more years need not be bridged with dormancy. Successful ATs have been shortened to few months from years ago (Early Breast Cancer Trialists’ Collaborative Group 1992). This implies that even in large studies no relevant risk by METs could be demonstrated that would have been initiated by dTCs in the previously longer treatment phase. Therefore, also an ectopic evolution of TCs in niches should not be a relevant step in the MET-P (Husemann et al. 2008; Hunter et al. 2018). Thus, only the risk of MET initiation by TCs of the three sMETs has to be considered (Fig. 2A).

### True local recurrences

True LRs are not considered a source of MET because 39.2% LR after breast conserving surgery did not result in a higher mortality in the seminal NSABP-B-06 study (Fisher et al. 2002). The high 4:1 risk found in meta-analyses has meanwhile been reduced to the subset of pN + findings (Early Breast Cancer Trialists’ Collaborative Group 2005, 2011a). Such a constraint is not plausible because survival curves show a continuously increasing MET-risk (Fig. 2D, E). This can be also shown by data of the MCR: 38.7% LRs occur in the subgroup of non-irradiated patients without any effect on survival, which reproduces the results of the NSABP-B-06 study with today’s data. Also, multifocal PTs did not show an increased MET risk (Pedersen et al. 2004; O'Daly et al. 2007).

### Positive lymph nodes

Synchronous pLNs are not involved in MET seeding (Ullah et al. 2018), nor has any robust data for cascading initiation been presented to date (Cady 1984; Engel et al. 2012). Long-growing PTs can infiltrate 10 or more LNs. If after a sentinel extirpation a LN recurrence is diagnosed it is often a singular pLN, which does not infiltrate the subsequent LN network like PTs. As mentioned above, METs are also initiated when no LNs are involved (Fig. 2D). In none of more than 10 solid tumors radical LN dissection has resulted in a survival benefit and can also be logically deduced from Fig. 2D (Engel et al. 2019, 2006; Giuliano et al. 2017). Survival curves in Fig. 2D, E show that the additional MET-risk decreases with each additional pLN. Therefore, pLNs are dead ends within the MET process even though animal experiments suggest a cascading spread is possible (Lambert et al. 2017; Pereira et al. 2018). The more than a century old hypothesis of cascade-like spread cannot be sustained (Halsted 1894; Crile 1906).

### Metastases

METs disseminate TCs with prognostic and predictive relevance (Cristofanilli et al. 2004; Mamounas et al. 2020). However, cascade-like initiation is clinically difficult to detect because, due to growth periods, patients would generally not experience METs initiated by METs. However, there are studies which claim that sMET can initiate tertiary METs. Estimates of initiation and growth of METs are usually not considered. In addition, any study must concede that “we cannot formally exclude an alternative explanation for the observed patterns, that each of these METs has seeded from an undetected subclone in the PT” (Gundem et al. 2015). This risk can also be largely ruled out because multiple MET in different organs and their segments are often genetically different (Yachida et al. 2010; Ramaswamy et al. 2003). MET surgery data also points against a MET-risk because, after R0-MET-resections, no new proximal METs limit the successful local resection as in 60% LR (inclusive multifocal LR) PTs without irradiation (Hölzel et al. 2010). Moreover, PTs infiltrate regional LNs of the respective organ of origin; this type of infiltrative behavior has not been observed from METs. Taken together, this data supports the assumption that LRs, pLNs, and METs most likely cannot initiate new METs. Therefore, all sMETs and also 2ndPTs of at least the next 10 years are already prevalent at the time of PT diagnosis (Fig. 1).

## Growth duration

The mechanisms of tumor growth remain largely unknown. From the smallest clusters of TCs to angiogenesis in the more advanced disease phase and thereafter, tumor growth varies due to differential cell divisions inherent to the molecular subtypes and unknown apoptotic rates. In addition, there are dependencies for extravasation and colonization on the tumor microenvironment (Weiss 1992). Nevertheless, growth duration can be estimated based on the initiation period (Fig. 2B), prevalence, PT- and MET-free durations, and can be transformed in volume doubling times (VDT). The growth of tumor foci can be described by growth trajectories (Spratt et al. 1993) that show the increase in the cell number over time (Fig. 2H). The growth will be mostly exponential. With the logarithm of the number of TCs, a straight line is obtained with an assumed asymptomatic logistic growth at the beginning and at the end. For a cohort, the age distribution of occult METs at the time of diagnosis and their occurrence during the course of the disease can be elucidated.

### The growth of PTs

There are three approaches to PT-growth: estimates from screening data showed a median VDT for women aged 60–69 years between 10 and 20 mm within 143 days (Weedon-Fekjaer et al. 2008). With these growth rates starting from a diameter of 2.5 mm (pT1a) the variability of the VDT with 25%/50%/75% percentiles of 65/143/308 days results in a growth time between 2.5 and 15 mm (pT1c) in 1.4/3.0/6.5 years (Fig. 3A). The 25% percentile at 1.4 years is consistent with approximately 25% that occur as interval cases in a biennial screening (Houssami and Hunter 2017). Since sMETs are rare in pT1a-PTs, it follows that all sMETs usually grow faster than PTs. The growth time of HR + occult BCs, which can be influenced by hormone replacement therapy, are correspondingly longer. The mean age at diagnosis 60.9/61.7/64.6 for pT1a-b/pT1c/pT2 also largely reflects PT growth. The variability of VDT is also apparent for molecular subtyping (Zhang et al. 2017).

The second approach is provided by prevention studies. With the above VDTs of 65/143/308 days and 32 VD, a pT1c-PT would grow from the first TC to diagnosis 5.7/12.5/27.0 years. In keeping with this, prevention studies show a continued reduction in incidence even 15 years after the end of a 5-year endocrine chemoprevention (Cuzick et al. 2015; Cuzick 2017). Fifteen years of PT growth corresponds to 171 days of VDs for 32 cell divisions. If the above 143 days are assumed this would result in a median growth of 12.5 years. That means that at PT diagnosis most of contralateral 2ndPTs for the next 12.5 years are already prevalent. In about 2% of cases synchronous PTs are detected. At the beginning of the mammography screening recommended for women aged 50 and over, 4% PTs are not yet detectable but already prevalent, and will occur in the next 12.5 years. The short-term increase of incidence by postmenopausal estrogen plus progestin therapy and its decline after weaning can also be explained by the prevalence and growth stimulation of PTs (Chlebowski et al. 2009, 2003; Engel et al. 2020). The third approach is provided by the estimation of MET growth from MET initiation to PT diagnosis.

### The growth of METs

The growth of the PT is also a chronometer for simultaneously growing MET (Fig. 2B). The growth of METs can be estimated if the 4 reference points are observed (Fig. 2B): a lower limit for initiation starting at about 1 mm PT, the timing of PT and MET diagnoses and the tumor-related death. The growth time of METs consists of the growth up to PT diagnosis and the MET-free time afterwards. In the special case of primary MET the MET has grown parallel to the PT (Fig. 2H). If METs were initiated at 2.5 mm, they would grow a median of 3.0/4.1 years parallel to pT1c/pT2-PTs and therefore must have more genomic differences to the PT than late-initiated METs (Bertucci et al. 2019). In pT2/pT3/pT4-PTs about 4%/12%/27% are primarily metastasized. In the case of T-N-M0-PTs the distribution function of the MET-free time with a follow-up of more than 20 years results in about 30% METs > 10 years and a median of 6 years (Fig. 3B) (Pan et al. 2017).

If T-N-M1 cases are also included, the distribution begins with a step corresponding to the proportion of M1 in all courses of disease with MET (Fig. 3B). A significant regression HR + PTs results with the Gompertz function y(%) = 98.9*exp(− 1.82*exp(-0.19*t)) (t(years): 0 -– 25). The median MET-free times including T-N-M1 result in 2.0/4.8 years for HR neg/pos PTs or together 4.4 years. The double is 8.8 years or 100 days for 1 VD and a plausible estimate for a median MET growth. The MET growth varies similar to PT growth. The MET-free and the post-MET time differ by a factor of more than 2.4 solely based on the PT HR status (Fig. 3B, C). But there is also a great variability within these subgroups. If the MET-free time is divided into quintiles, then the 5-year post-MET survival is 10%/40% for the outer 20% limit values of approx. < 1.5 and > 11 years (Fig. 3E) (Hölzel et al. 2017a). The longer the MET-free time the more favorable the prognostic factors of the PTs are.

Also, this contradicts a continuous MET initiation from dTCs after R0 removal. METs that would not be initiated until 5 or 10 years after PT diagnosis would have to grow very fast and most of them would have to originate from triple negative PTs, which is not observed. Growth rate variability reflects the distribution of event-free times, which are positively skewed with a long upper tail for METs that occur without dormancy after 15 years or more. Despite the increase in MET frequency with TD, MET is an autonomous process within molecular subgroups. Larger PTs do not initiate more aggressive METs. According to this principle, METs initiated early or later on grow comparably fast (Figs. 2H, 3C).

### The growth of LRs

Late initiations of true LR occur immediately before PT removal and are likely to have the longest LR-free times. The growth period of true LRs can be read from the breast conserving surgery studies with and without radiation (Early Breast Cancer Trialists’ Collaborative Group 2005) and is about 6 years (Fig. 3E) because thereafter the slope of the cumulative incidence of LRs is the same in both groups. The earliest initiations of true LRs are 18% multifocal PTs and without them the median LR-free time is approximately 2.5 years and independent of follow-up (Fig. 3E), while median ipsilateral 2ndPT-free time increases to half the follow-up time due to continuous initiation of PTs (Fig. 2H) (McGrath et al. 2010; Smith et al. 2000). Six years of growth results in a VDT of about 68 days and 32 VDs, almost 2.1 as fast as that of the PTs.

### The growth of pLNs

The growth of pLNs cannot be easily estimated because of the lack of robust data on pLN-free time due to successful ATs and LN dissections. According to data of the MCR in 100 patients with pT1b-BC (7.5 mm), 13% pN + and a total of about 36 pLNs are to be expected. If PTs reach a diameter of 15 mm after about 14 months, 42 more LNs are infiltrated, in total 25% pLNs. In today's serial sentinel preparation, small PTs have about 12% macro-METs and almost as many isolated TCs and micro-METs (Colleoni et al. 2005; Boer et al. 2009). If the 12% newly infiltrated LNs had a size of 0.2 mm at 7.5 mm (border between ITC and micro-MET) and then grew to a size of 2 mm, this results in a VDT of 43 days, 3.3 times that of PTs (Engel et al. 2012).

## MET-free, pre- and post-MET survival

The survival time of a MET-related death is composed of two periods of MET growth (Fig. 2B), the pre- and post-MET survival, the former as the sum of time to PT diagnosis and MET-free survival. Since MET diagnosis does not change the growth, the ratio of pre- and post-MET survival will vary little. Figure 3E shows a post-MET survival of approximately 2 years and because the time to MET was estimated at 8.8 years the ratio is 4.4. Since median post-MET survival reveals the influence of various growth factors, for example, MET-free survival or KI67 between 0.7 and 3 years in Fig. 3C, the ratio 4.4 can be used to estimate the growth duration of MET from post-MET and the initiation by the PT (Hölzel et al. 2017b).

This is not inconsistent with the variability of MET-free survival. The longer a PT disseminates, i.e. the larger the PT, the longer the occult MET growth and the shorter the MET-free survival time. This also applies to pLNs. Of all METs 35% occur at 0pLNs. The MET-risk increases when micro-METs are already detectable in LNs and it doubles with the first pLN (Fig. 2D) (Engel et al. 2012; Colleoni et al. 2005; Boer et al. 2009). If more LNs become positive these occult METs continue to grow, the MET-free intervals become increasingly shorter. In 0/2/8–10 pLNs, primary METs are diagnosed in 0.7/4.1/14.2%. The number of pLNs is the most important clinical prognostic factor, it is a chronometer for MET but not its cause (Engel et al. 2012; Giuliano et al. 2017). Therefore, evidence for extensive LN-dissections for MET risk reduction is lacking in the most common solid tumors but do not contradict LN extirpations for regional control. This is also true for LR with early dissemination and parallel LN and MET initiation. Again, it follows that LR and pLN should not be the cause of MET, because otherwise not shorter but longer MET-free survival would be the consequence.

## Principles of tumor growth and the MET process

The available evidence from well-known data on PT findings, age-related incidence, and the course of disease provide a plethora of facts that can be summarized into five principles on the initiation and growth of PTs and METs. Neither initiation of METs by secondary foci nor a long-term delay through dormancy have been convincingly shown so far. This indicates a (Valastyan and Weinberg 2011) pre-diagnosis MET initiation principle of the primary tumor. Growing PTs continuously initiate life-threatening METs. With every millimeter of a growing PT the proportion of METs increases (Fig. 2E). This random process leads to variability in the age of the MET at diagnosis and the MET-free time, which together give a mean duration for MET growth of about 8.8 years (Fig. 2H). This is a (Hanahan and Weinberg 2011) growth-dependent metastasis principle.

Growth rates of PTs and their METs can each differ by a factor of 10 and more with a long upper tail (Fig. 3A). Despite the great variability, the dissemination of TCs and initiation of pLNs and METs, depending on TD, are likely comparable. The processes only run at different speeds, which is a (Yates et al. 2017) time lapse principle. Therefore, the properties of 25% interval PTs are comparable to populations without biennial screening despite their rapid growth (Houssami and Hunter 2017), or early advanced PTs are not predominantly triple negative PTs.

Dissemination, acquisition of MET-competence or growth rates are associated with prognostic factors. HR or HER2 status define subgroups in which the principles apply and are the first targets for personalized therapies. Present and future clinically subgroups will allow a tuning of therapeutic strategies and be the basis of progress, (Lambert et al. 2017) a subgroup tuning principle against MET.

Triple negative or luminal A-PTs usually do not initiate contrary METs. That is, the relationship of the growth rates of PTs and their METs varies only slightly (Fig. 3F). The relationship must be greater than 1 because there are M1-PTs and it cannot be 10 because then there would be mostly M1-PTs. The estimate of about 8.8 years of growth up to MET and 12.5 years for PT results in a ratio of 1.4 and with 8.8 pre- and 2 years post-MET growth of 4.4. The growth relations can be seen as a (Peinado et al. 2017) robust PT/MET growth and pre-/post-MET survival ratio principle. The tumor-specific long-term survival supports these principles and they are a challenge for gene expression tests (Fig. 3G) (Colleoni et al. 2016).

## Results on the effect of adjuvant and neoadjuvant treatments

All sMET are prevalent at primary diagnosis but all MET are life-threatening. The principles of MET-P explain successes and limitations of ATs and resulting shape and change of survival curves. Two questions are relevant to successful ATs: 1. How does treatment efficacy relate to MET properties such as size, type, and localization? 2. How long does treatment have to be given? These questions are also to be asked about occult 1st-2ndPT.

### Eradication of primary tumors

Complete pathological remissions of over 50% can be achieved with neo-ATs in a few weeks (Cortazar et al. 2014; Robidoux et al. 2013; Minckwitz et al. 2012). Endocrine therapies achieve a less complete remission, but downsizing and prolonged localized control of the PT are possible (Fontein et al. 1990; Fentiman et al. 1990; Spring et al. 2016; Ellis et al. 2017). The lower efficacy of endocrine neo-ATs depends on the size of PTs, because the reduction of the incidence by endocrine chemoprevention in studies is initiated after a short delay of about 3 VDs, which results from the inclusion criteria “negative mammography”. (Cuzick et al. 2015; Fisher et al. 2005). Thus, prevention eradicates PTs quickly, especially under aromatase inhibitor (Cuzick 2017; Cuzick et al. 2020). During a 5-year preventive therapy new PTs may develop. Even these small PTs grown for a maximum of 5 years are eradicated at 50%, since the reduced incidence does not change for 15 years. That is, prevention successfully eradicates PTs of all sizes. However, the properties of the 50% eradicable or resistant HR + PTs are not known. Therefore, endocrine ATs have two effects, eradication of MET and contralateral PTs. Regarding the duration of treatment, many studies show that prevalent contralateral PTs are eradicated after 1 and 2 years. (Early Breast Cancer Trialists’ Collaborative Group 1998).

### Eradication of metastases

Overt METs currently remain resistant to treatment. This also applies to nearly detectable MET, because in clinical trials with (neo-) ATs the survival curves of MET-free time do not separate in the first months (Early Breast Cancer Trialists’ Collaborative Group 2011b, 2012; Cameron et al. 2017). Thus, there is a size-dependent upper threshold for effectiveness. A lower limit is not distinguishable in our data because successful ATs have achieved a uniform eradication of even the smallest METs (Fig. 3G).

This also follows from the distribution of the number of pLNs: the fraction with 0–2 pLNs is largely independent of the TD (Fig. 2E), but the mortality increases with each additional pLN (Fig. 2D). In these subgroups, there are a similar number of small METs which can be destroyed by ATs. This explains the more than 10% improvement in survival seen in the past decades, regardless of the PT size (Hölzel et al. 2017a; Welch et al. 2016). With a favorable prognosis (0pLN, pT1), the relative 5-year survival today reaches almost 99% (Fig. 2C-G) (Noone et al. 2021).

A selective eradication of early initiated METs with few mutations or late-onset MET due to smaller foci are not recognizable (Fig. 3H). However, the ATs of the past few decades provide an organ-specific MET eradication: Nowadays, METs in bone or lung are about 50% or 30% less frequent, whereas there is no evidence for a relevant eradication of liver and CNS METs until now. Such successful ATs produce a paradoxical effect. If the early bone METs become less frequent and the progression begins with the later CNS METs, then MET-free survival becomes longer and post-MET survival shorter (Hölzel et al. 2017a; Jurrius et al. 2020). In contrast, the MET pattern in T-N-M1 (at diagnosis) has not changed in the last decades because it is untreated and mirrors the biology of BC (Hölzel et al. 2017b). A rationale for the duration of the previously known ATs is not evident.

The MET-P can also be explained by the effects of delays (Hanna et al. 2020). If surgery is postponed, further METs and pLNs are initiated with each additional millimeter of TD. This is the contrary of the logic of screening to shorten the duration of the dissemination of the PTs and thereby prevent METs (Fig. 2B, C). When ATs starts delayed, METs continue to grow in the interim and fewer are eradicable later (Figs. 2H, 3B). But for the remaining METs, AT is effective, for HR + PTs even after many years of delay. (Delozier et al. 2000; Veronesi et al. 2010). That also suggests the effectiveness of short treatment durations.

#### Eradication of LR and pLNs

The eradication of occult LRs by irradiation is excellent. Radiation therapies even made breast conserving surgery possible due to its efficacy in faster growing LRs compared to PTs which are also prevalent in the form of ipsilateral multicentric 2ndPTs (Fig. 3E) (Fisher et al. 2002). Therefore, ATs may improve local control of multifocal and multicenter LRs, the different origins of which should be shown by their clonality (Kim et al. 2018).

Regional control of pLNs is achieved with today's surgical and neo-ATs making a LN relapse in < 5% during a time span of 15 years a rare event. Survival is optimal if no residual tumor remains after neo-AT primary and in the LNs (Cortazar et al. 2014; Minckwitz et al. 2012). Thus, ATs also contribute to regional control because in the absence of an axillary dissection they act as neo-ATs on not removed LNs with isolated TCs and micro-METs (Giuliano et al. 2017; Mamtani et al. 2017).

#### Principles of neo- and adjuvant treatments

Treatment cohorts of MCR and clinical studies suggest also five AT-principles: (Valastyan and Weinberg 2011) Endocrine chemoprevention can eradicate about 50% of 1stPT and 2ndPTs, regardless of whether the PT develops during the therapy or is already prevalent. (Hanahan and Weinberg 2011) 50% complete pathological remissions are possible with neo-ATs. (Yates et al. 2017) Neo- and adjuvant therapies can eradicate organ-specific METs of all sizes up to detection limit. (Lambert et al. 2017) The eradication of PTs and METs is achieved in a short time and suggests the challenge “how short is short enough”. (Peinado et al. 2017) In form of a hypothesis: The rapid eradications make intrinsic resistance in PTs and METs more likely than a therapy-induced resistance (Razavi et al. 2018).

## Discussion

These principles described above are evident in current treatment schemes. PT growth and the effects of AT can be used, for example, to estimate the risks of postponing screening or surgery. If a biennial screening for 100,000 women aged 50–70 years is postponed by one year, about 64 additional deaths are expected after 15 years (in press) However, the principles also illustrate the limits of ATs and the necessary further development of prognostic and predictive factors. In particular, the gradual progress in median post-MET survival of about 11 months and the increasingly expensive treatment costs over the last 3 decades is an ongoing challenge. (Munich Cancer Registry 2021; Vivot et al. 2017) The duration of successful treatments is an important issue to consider in this discussion. If there is no rationale for the duration of a treatment regimen, even with available study data an overtreatment is likely, but undertreatment is also conceivable (Goldhirsch et al. 2013). Optimizations in the sense of de-escalation “How short is short enough” (Peto 1996; Smith et al. 2014) are ethically problematic, scientifically unattractive, and usually economically disadvantageous- in contrast to “how long is long enough”. But there are such studies that have achieved equal efficacy with shorter treatment durations (Pivot et al. 2013; Petrelli et al. 2020). Real world data may help to examine possible improvements, for example, in the case of good results despite deviations from guidelines. Other examples include the use of gene expression-based recurrence score and waiving of ATs in certain populations or the evaluation of alternative decision rules when a recurrence score is retrospectively obtained according to follow-up status (Gnant et al. 2015; Veer et al. 2017; Zhang et al. 2020; Andre et al. 2019; Walter et al. 2020). If growth of PTs varies by a factor of 10 or more, new biomarkers for the duration of therapies are conceivable as a further step towards personalized medicine and could be tested with registry data (Anandan et al. 2020).

It is particularly difficult to change knowledge or treatments acquired with studies. Five years of chemoprevention or the hormone replacement therapy are examples. There is no evidence that HR + PTs can be initiated and grow to detection within 5 maybe even 10 years. Prevalent PTs grow faster and are implicated in upstaging at diagnosis. The risk of additional PTs can only be detected in long-term follow-up. Another example is extended endocrine AT (Davies et al. 2013; Curigliano et al. 2017; Burstein et al. 2019; Early Breast Cancer Trialists’ Collaborative Group 2022), which also contradicts above principles. Again, the studies provide correct results, but their interpretation may need to be reconsidered. If AT is prolonged even after 5 years, 50% of MET occurred. But 50% are still prevalent and benefit. However, if 5 years of AT is continued, there is no immediate effect. Endocrine therapy eradicates occult METs on the one hand and prevalent PTs and their future METs on the other. With one short AT and intermittent short preventive therapies needed because of new PT arising throughout life, the treatment time could be reduced to at least 70%. This also reduces side effects and their complex management (Engel et al. 2020b). When a therapy has 2 effects the evidence from studies should be questioned. Further conclusions from the principles arise for LN dissections and after-care. Despite many studies, pLNs are not a cause of MET and therefore extended LN dissections cannot be justified. Also, the efficiency of diagnostics in aftercare cannot be shown because all sMET are already prevalent.

Impulses can also give the question, of what characteristics PTs or METs have that have been eradicated or have remained resistant (Musgrove and Sutherland 2009). In the case of organ-specific efficacy, success is primarily linked to the microenvironment. CNS and liver are inaccessible and pharmacokinetic and molecular mechanisms are discussed (Minchinton and Tannock 2006). Early HR + , contralateral 2ndPTs that arise despite endocrine AT of the 1stPT would have to show differences from HR + 1stPTs in the genomic landscape. This question should also be asked about METs that can only be partially eradicated. The rapid eradication of PTs (Cortazar et al. 2014; Cameron et al. 2017) and METs (Conte et al. 2018) within 1–2 VDs suggests that a non-responsive tumor is more of a consequence of intrinsic resistance mechanisms than of acquired ones (Holohan et al. 2013; Jeselsohn et al. 2015). Meticulous observations, laboratory and mathematical modeling can help clarify hypotheses in advance and can perhaps accelerate innovations. This is supported by the hope that complexity is a manifestation of only a few fundamental principles (Hanahan and Weinberg 2011).

## Conclusion

Clinical trials and real-world data with lifelong follow-up and progression-free and post-progression survival elucidate dissemination of TCs, initiation and growth of sMETs as well as PTs. Few principles describe tumor growth and the MET-P and form a framework for developments. Dormancy and stepwise MET initiations by sMETs are not supported by real-word data. Also, principles about neo- and ATs can be derived from data about successes and limits of preventive, diagnostic and therapeutic interventions. Tumor growth and successful changed MET patterns question the duration of preventive and adjuvant therapies.

Primarily, real-world cancer registry data should clarify how findings, treatments, or MET pattern change and which medical advances have a relevant impact on a population. With quantitative models (Altrock et al. 2015) and comparative effectiveness analyses (Hershman and Wright 2012; Concato et al. 2010) principles about tumor growth and therapies can be derived and validated. Feedback from these data analyses can support delivery of care and improve its quality, especially when many outcome parameters are compared. Impulses for health care-relevant translational research can be given. Linking cancer registry data to biomaterial and sequencing data could accelerate interdisciplinary knowledge acquisition. Such expansions of the systematic use of available data can become a vast source of knowledge.

## Supplementary Information

Below is the link to the electronic supplementary material.

Supplementary file1 (DOCX 1120 KB)

## Data Availability

The data of the tumor registry are updated daily. Extensive evaluations are regularly prepared for hospitals on their own data. Updated and supplementary data are made available to readers upon request.

## Abbreviations

AT: Neo-adjuvant systemic therapy
BC: Breast cancer
HR: Hormone receptor
HRT: Hormone therapy
(s)MET: (Secondary) metastases, LR, pLN, distant-METs
MET-P: Metastasis process
LR: Local recurrence
(p)LN: Positive lymph node (locoregional)
(1st/2nd)PT: (First/second-contralateral) primary tumor/primary breast cancer
(d/c)TC: (Disseminated/circulating) tumor cell
TD: Tumor diameter
VD(T): Volume doubling (time)
