# Supplementary material for: Breast cancer: emerging principles of metastasis, adjuvant and neoadjuvant treatment from cancer registry data
Source: J Cancer Res Clin Oncol. 2022 Dec 20;149(2):721–35. doi: 10.1007/s00432-022-04369-4 (PMC9931789; doi:10.1007/s00432-022-04369-4)
Supplement: Supplementary file 1 — Supplementary file1 (DOCX 1120 KB) [file 432_2022_4369_MOESM1_ESM.docx]

**Supplement: 17 Figures shown individually with a higher resolution**

# Figure 1 Initiation and growth of a PT and its secondary foci

Growing PTs can initiate LRs, pLNs and METs with different gene signature. They can be diagnosed synchronously with PTs (filled symbols), remain occult, will be eradicated by ATs or occur in the course of disease. The article and this figure were inspired by LR Yates et al (2017).


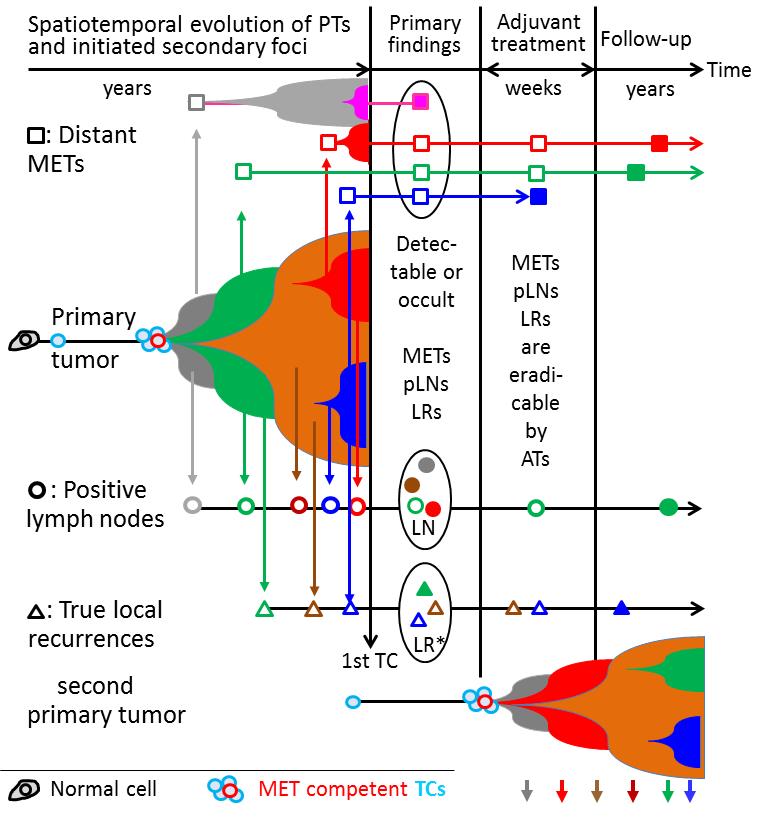


## Figure 2A Tumor cell dissemination and initiation of LRs, pLNs, METs

| 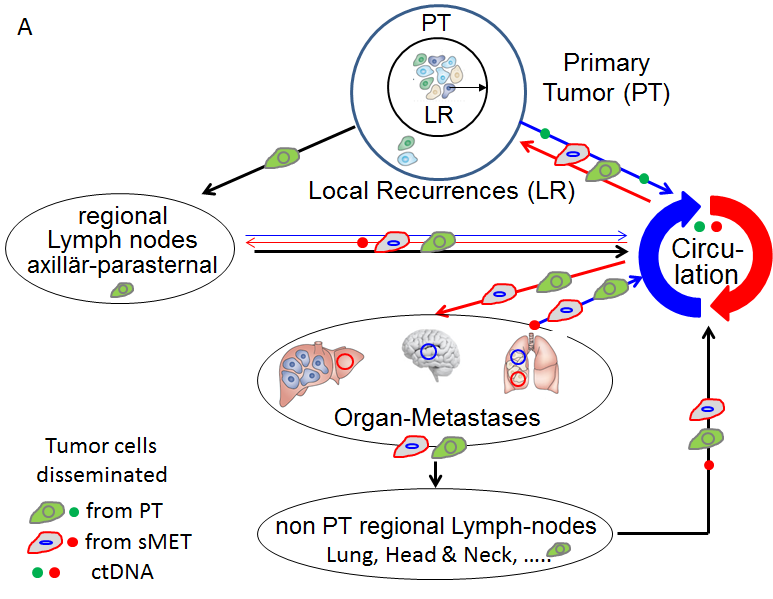 |
| --- |

## Figure 2B Tumor size, dependent MET and pLN initiation and survival

**I**: METs initiation until the removal of the PT. **II**: PTs will initiate up to pT1c about 10% METs, 1.5% are already T-N-M1. **III**: During growth from pT1c to pT2 the METs already initiated continue to grow amd 4.5% become T-N-M1, 25% METs and 20.3% pLNs are additionally initiated which can be avoided by early detection at pT1. Equal rapid MET growth is outlined. Because of successful ATs, the percentages depend on the reference period and can therefore vary.

| 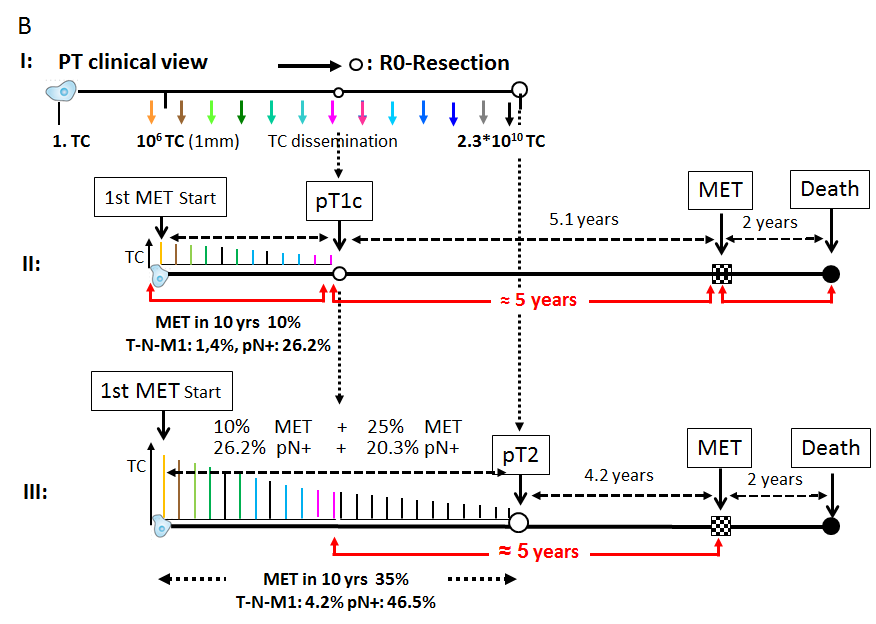 |
| --- |

## Figure 2C Relative* survival depending on tumor diameter for T-N-M0 PTs. In the MCR catchment area, there has been a screening program for women between 50 and 70 years of age since 2006, in which 50% participate.

*The relative survival is an estimate for tumor-specific survival and is calculated by dividing the overall survival after diagnosis by the survival observed in the general population with comparable age distribution.

| 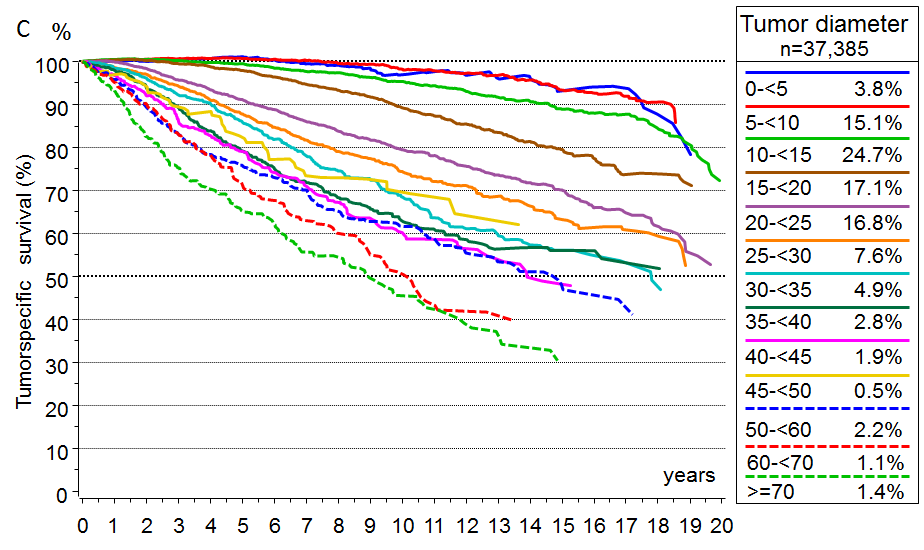 |
| --- |

## Figure 2D Relative survival depending on the number of pLNs. The arrows are the same length. The length corresponds to the mortality of 20% in 0pLNs after 20 years. The arrows show the decreasing risk of the increasing number of pLNs.

| 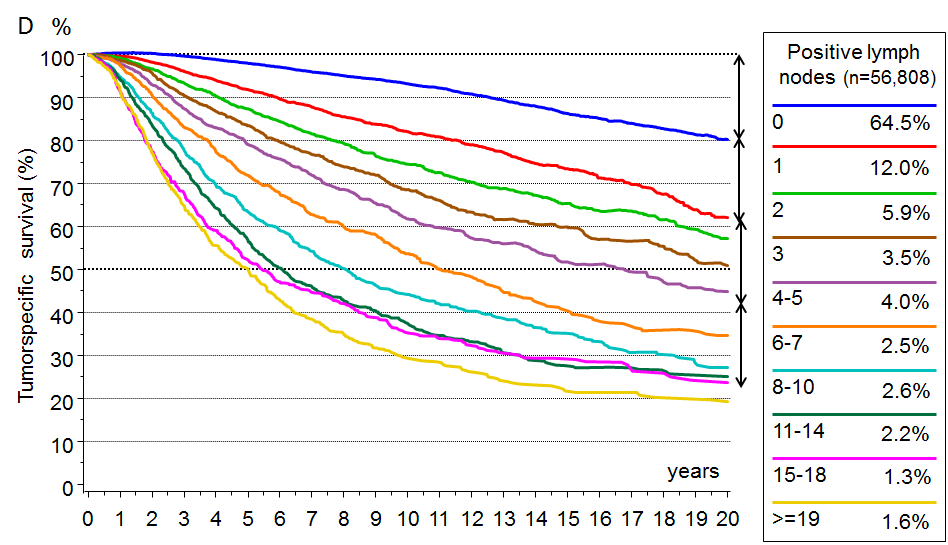 |
| --- |

## Figure 2E Distribution of the number of pLNs in dependence on tumor diameter (n=30,170). Cumulative observed data (dotted lines) and fitted Gompertz functions (Gf) (solid lines) show for each TD the percentages with >npLNs. For 0pLN results: y (% ≥0pLN) = 100 – 75.4*exp(-2.82*exp(-0.063*TD)) (TD: 2 – 90mm) The dotted black line connects the relative 15-year survival in each subgroup with 0pLN and tumor diameter and the solid regression line estimates the proportion with occult MET (0pLN*). At 45mm, 37.1% are still 0pLN, and at least 12.1% already have initiated MET. This means 25.9% will still be MET free after 15 years, a 15 year survival of 69.8%.The black line is the fitted Gf for the observed tumor specific 15-year mortality (diamonds) depending on TD. The stacked barplot visualizes the distribution of pLNs at 22.5mm TD. (Quelle: Engel j et al: Lymph node infiltration, parallel metastasis and treatment success in breast cancer. Breast 2019 Vol. 48 Pages 1-6) 45mm 37.1 25.9

| 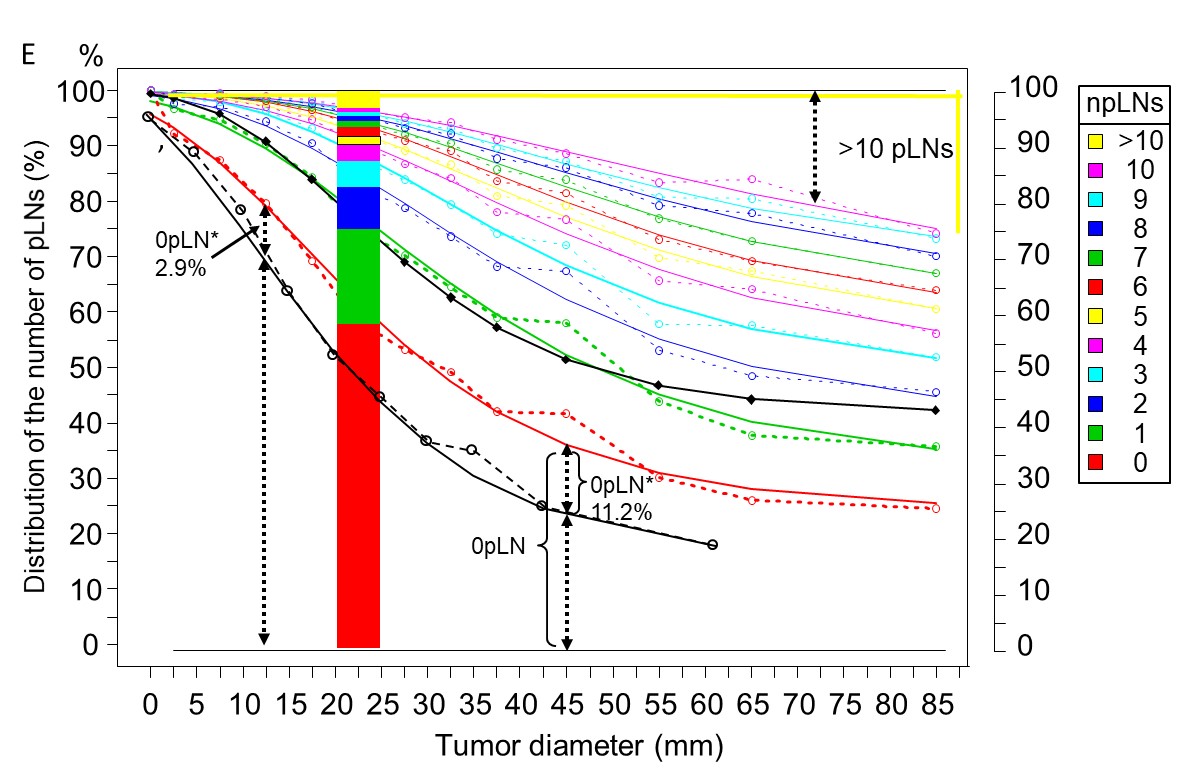 |
| --- |

## Figure 2F Overall survival after breast conserving surgery and irradiation

Overall survival for patients with pT1-2 PTs, breast conserving surgery and irradiation. The blue dotted and dashed subgroups are samples with the same distribution of age, pT and LNs as the subgroup without radiation (brown curve). Further division into the two subgroups with and without LRs showed no increased mortality risk from LRs.

| 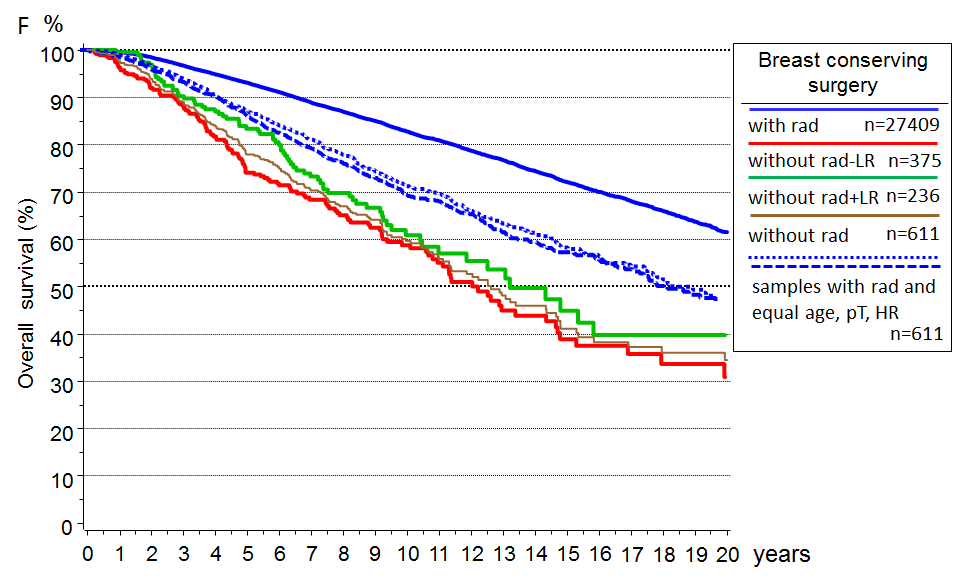 |
| --- |

## Figure 2G Trend: relative Survival depending on pT1-2

Relative survival for pT1c and pT2 PTs and 4 time periods from 1978. The improvement after 15 years is about 10% absolute in both subgroups

| 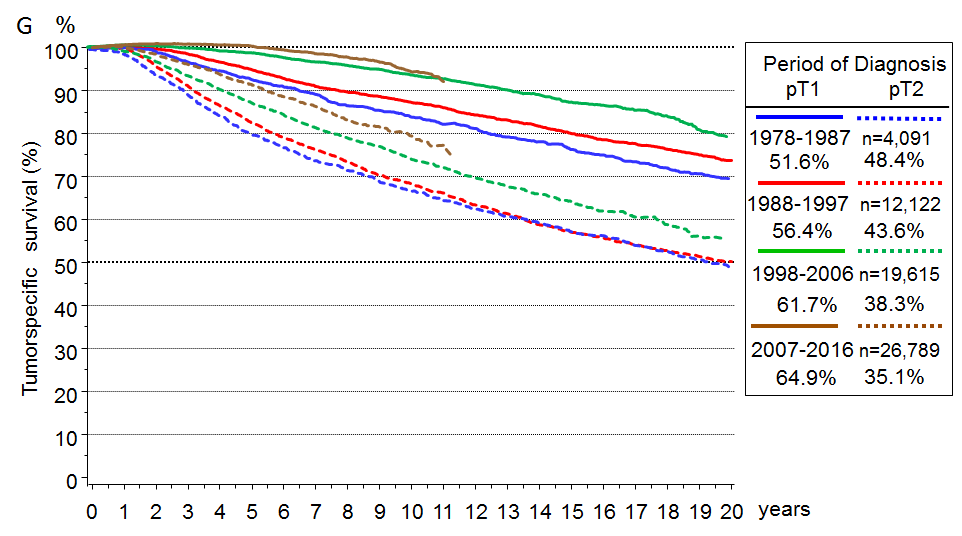 |
| --- |

## Figure 2H Growth trajectories

Growth trajectories for MET and 2ndPTs. Each trajectory describes the number of TCs (log scale) as a function of a median growth time (8 MET or 12 PT years) relative to the PT. The age of a MET or 2ndPT at the time of diagnosis of the 1stPT indicate the age scales. The sum of age and event-free survival is on average constant. Blue trajectories represent synchronous events, black ones for 2nPTs, which are initiated after PT diagnosis. The age distributions of METs are snapshots at the time of PT diagnosis and a delayed time afterwards. Since no new METs are initiated, there are no small METs for delayed ATs and the larger ones have already been discovered.

| 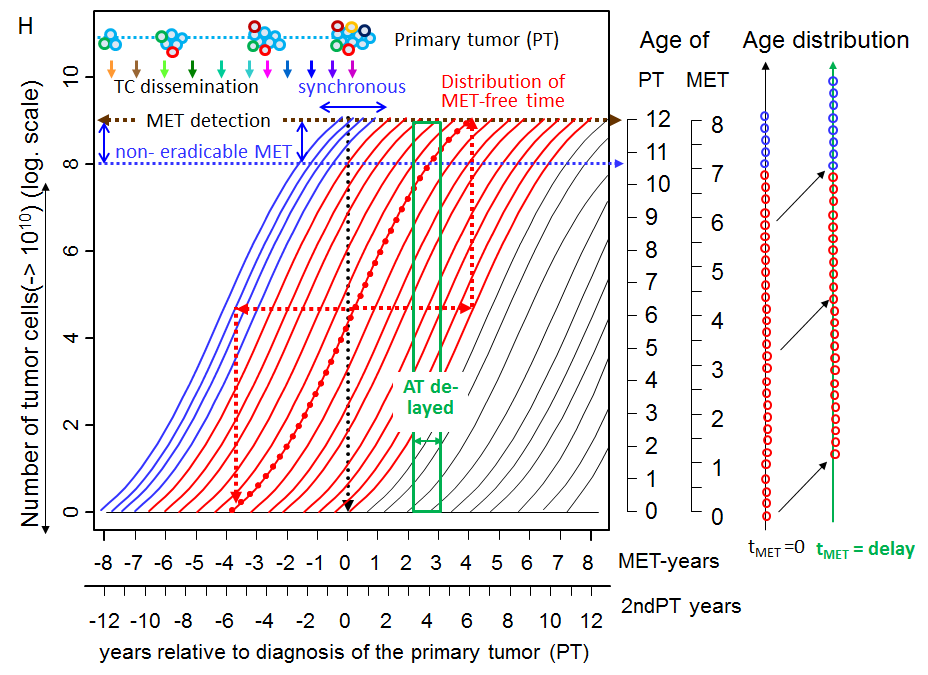 |
| --- |

## Figure 3A Volume doubling and tumor growth

Growth durations of PTs from 2.5mm depending on estimates of the 25% / 50% / 75% percentiles VDTs according to H. Weedon-Fekjaer*. The variability is illustrated by the growth times from 2.5 mm up to pT1c (15mm) or pT2-PT (28mm). (* H. Weedon-Fekjaer et al.: Breast cancer tumor growth estimated through mammography screening data. Breast Cancer Res 2008 Vol. 10 Issue 3 Pages R41)

| 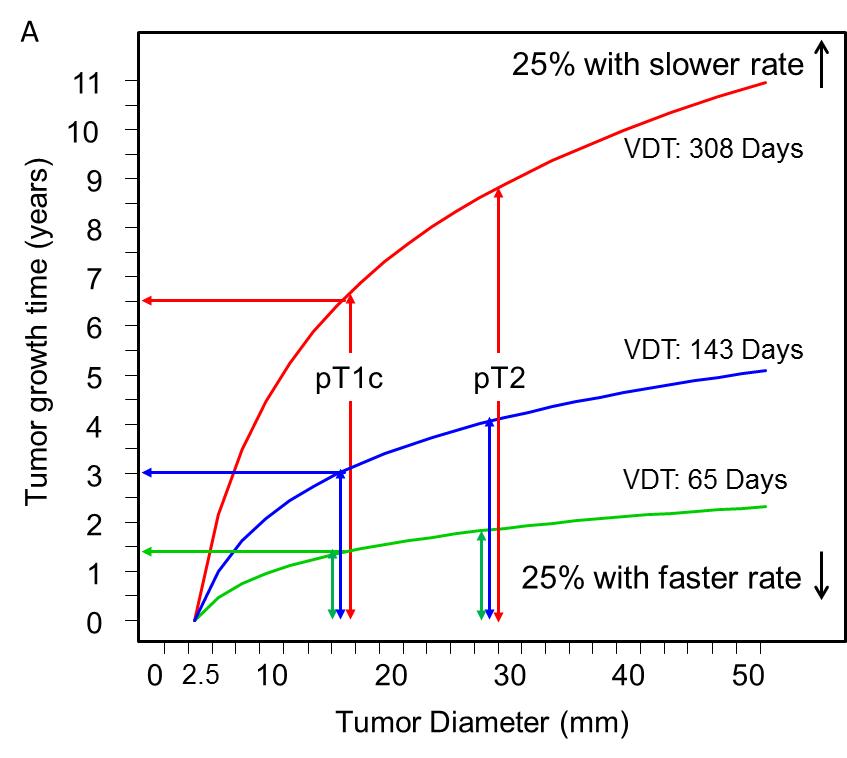 |
| --- |

## Figure 3B Hormone receptor dependent MET-free survival

Distribution functions of the MET-free times for T-N-M0 and HR+ positive (red) and HR- (blue) PTs from the time periods 1988-97 and 1998-2007. Because of the observation period of 20 years the distributions from 1998-2007 reach almost 100% at 20 years. About 30% of the MET appear after 10 years. The dashed lines also show the fraction of T-N-M1 for 1988-97.

| 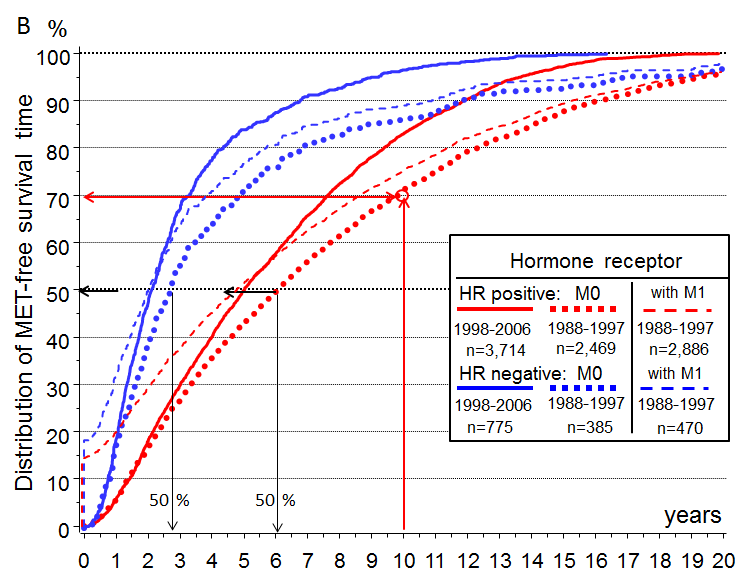 |
| --- |

## Figure 3C Cumulative incidence of METs and survival after MET depending on KI67. The median survival at T-N-M0 in the event of tumor-related death for the two outer subgroups is approximately 3.1 and 1.5 years. The post MET survival curves are covered at 22%.

| 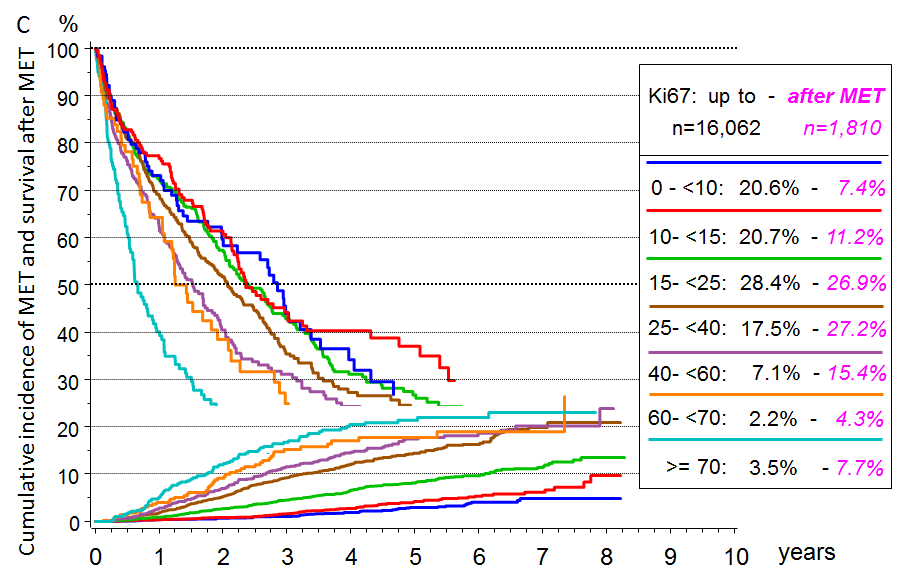 |
| --- |

## Figure 3D Cumulative incidence of LRs after breast conserving surgery with and without irradiation.

Black dotted lines are parallels and show that late LRs increases in both groups comparable to the incidence of contralateral 2ndPT. The dotted blue curve is similar to those for LRs without irradiation and is the fictive initiation of true LR after forwarding the twofold LR-free time of about 26 months.

| 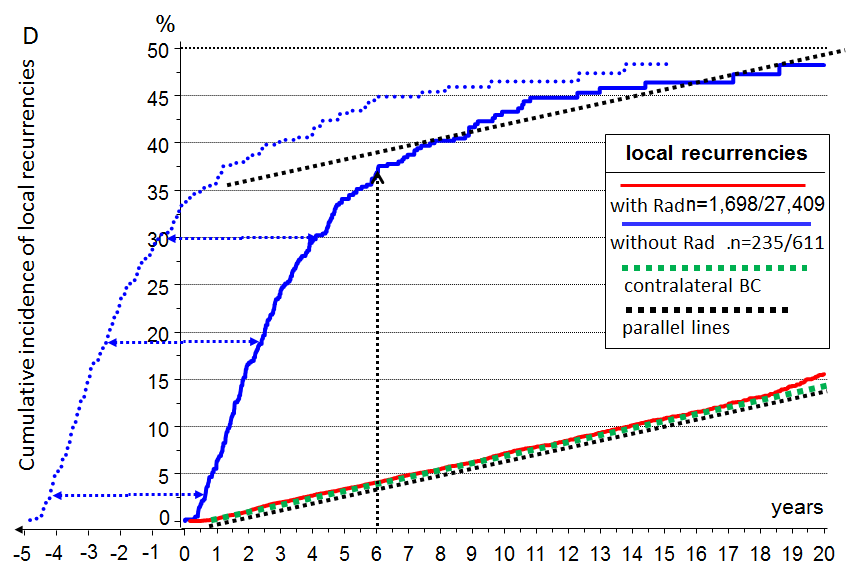 |
| --- |

## Figure 3E Overall survival from MET of HR+ PTs as a function of MET-free time

PTs are diagnosed between 1988-2006 with > 20 years follow-up and n=8,127 METs observed. The differences of PTs of the 1st and 5th Quintile (<1.5, ≥11 ys) are 12.4/36.9% for 5 years survival, 23.9%/45.7% for pT1, 18.3 / 42.4% for pN0 and 62.6 / 26.3% for grade 3.

| 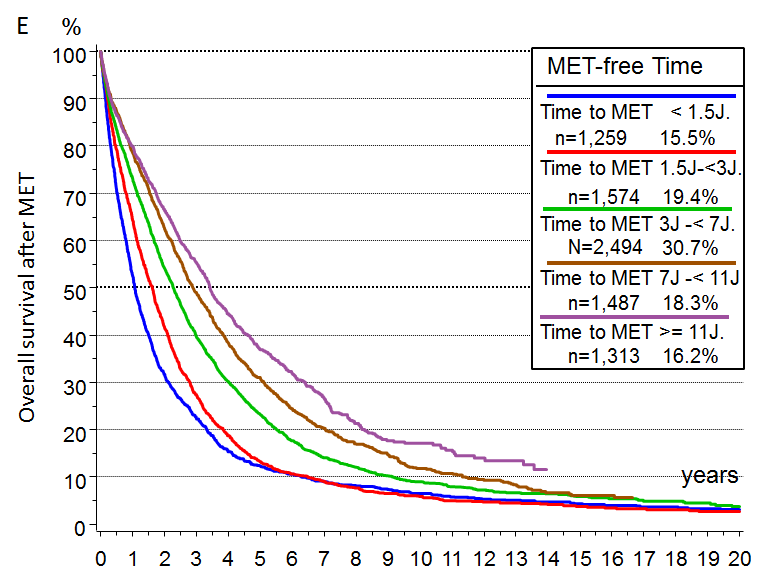 |
| --- |

## Figure 3F PT and MET growth relation principle

Relation of tumor growth of PTs and METs (pT1, pT2), MET-free survival and post MET-survival (red) at different growth rates

| 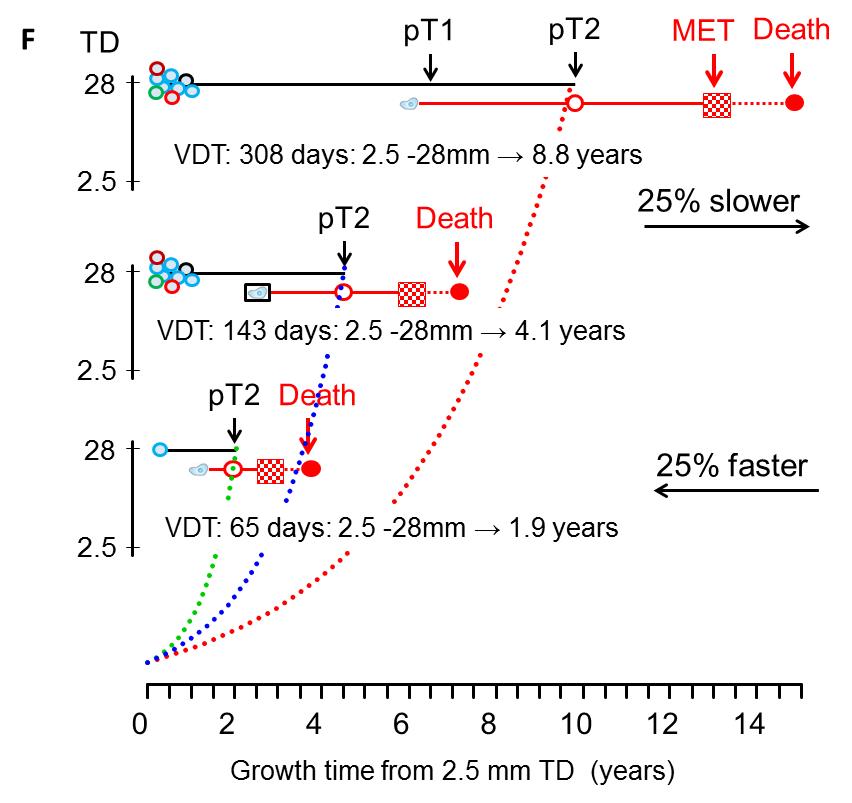 |
| --- |

## Figure 3G Relative survival of HR+ and HR- PTs.

| 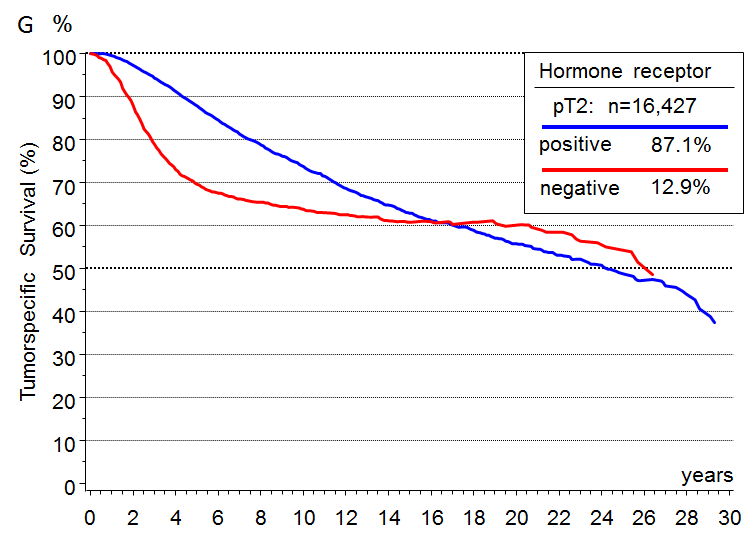 |
| --- |

## Figure 3H Time trend of bone-MET-free survival

Cumulative incidence of bone METs as a function of 4 time periods since 1978. (Kaplan-Meier method without concurrent risks) The black lines sketch fictitious selective eradication of early (dashed) or late (dottet) initiated METs alternative to green line.

| 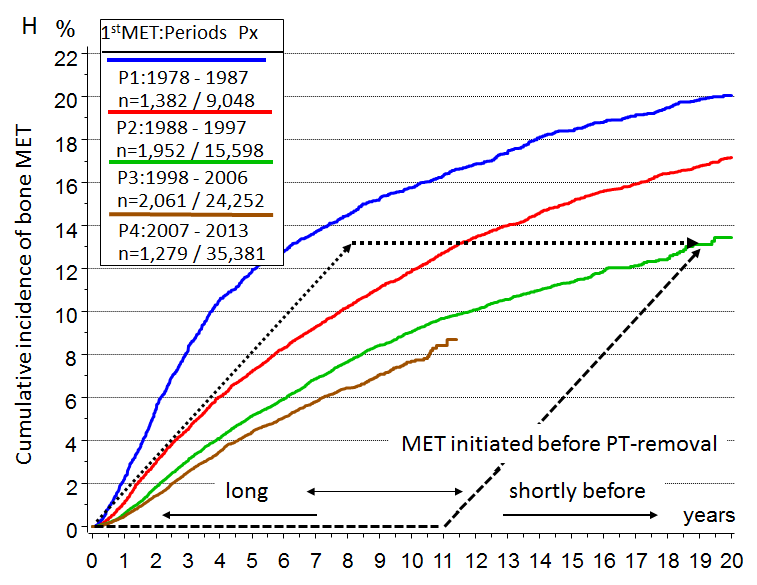 |
| --- |
